# F18 fluorodeoxyglucose uptake in progressive transformation of germinal centres

**DOI:** 10.2349/biij.4.1.e6

**Published:** 2008-01-01

**Authors:** B Rehani, Y Dowdy, A Bharija, P Strohmeyer

**Affiliations:** Department of Internal Medicine, Kettering Medical Center, Ohio, United States

**Keywords:** F^18^ FDG-PET/CT, Progressive Transformation of Germinal Centres (PTGC), Hodgkin’s disease (HD)

## Abstract

FDG-PET/CT is a widely established imaging modality for staging, restaging and monitoring therapy response in lymphoma patients. Progressive transformation of germinal centres (PTGC) is a benign condition presenting characteristically as asymptomatic lymphadenopathy. This paper presents a case of a 53-year-old man with a history of Hodgkin’s disease (HD) whose F^18^ FDG-PET/CT scan showed high uptake in left axillary lymph nodes (SUV 3.8). A subsequent, left axillary lymph node biopsy revealed PTGC. PTGC can present as a false positive finding on FDG-PET/CT in lymphoma patients and biopsy should be done in HD patients in clinical remission but have a positive FDG-PET/CT scan.

## CASE REPORT

**Figure 1:**
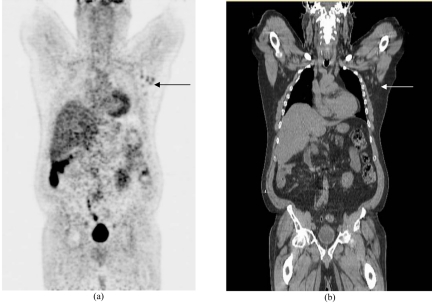
FDG PET-CT scan of the patient. Arrow shows small lymph nodes in the left axilla with the highest standardised uptake value of 3.8 and measuring less than 1 cm in size.

A 53-year-old man underwent FDG-PET/CT scanning for detection of recurrent disease. He was diagnosed with Hodgkin’s disease (HD) five years ago and successfully treated with chemotherapy. The patient was asymptomatic. 12.6 mCi FDG was injected and images acquired using a Siemens Biograph 6 PET-CT scanner (Siemens AG, Munich) (Figure 1). The PET/CT image showed small lymph nodes in the left axilla with the highest standardised uptake value of 3.8 and measuring less than 1 cm in size. FDG-PET/CT imaging has been found to have higher accuracy than FDG-PET and CT alone in staging and restaging of patients with lymphoma [1,2].

**Figure 2:**
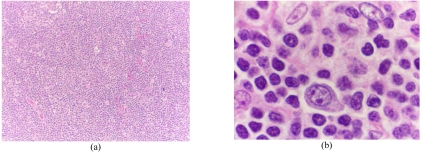
Photomicrograph of left axillary lymph node biopsy (a) shows progressive transformed germinal centre and loss of normal architecture (haematoxylin and eosin stain, x10); (b) shows small lymphocytes, histiocytes and immunoblasts (haematoxylin and eosin stain, x40).

Left axillary lymph node biopsy was performed and revealed progressive transformation of germinal centres (PTGC). Figure 2a shows progressive transformed germinal centre and loss of normal architecture, while Figure 2b shows small lymphocytes, histiocytes and immunoblasts.

## DISCUSSION

PTGC was initially described by Lennert and Muller-Hermerlink as large follicles composed predominantly of diffuse small lymphocytes and an obscured mantle zone [3]. PTGC is most commonly seen in lymph nodes in association with reactive follicular hyperplasia [4]. PTGC may precede lymphocyte predominant Hodgkin’s disease (LPHD) or can be present subsequently in lymph node biopsies of lymphoma patients as seen in this case; but the presence of PTGC is not associated definitively with an increased risk of developing HD [5].

The causes of false positive FDG-PET/CT include infection, inflammation, granulomatous disease [6,7] and immunisation [8]. However, the FDG uptake in PTGC has been rarely documented in literature [9] and can present as a false positive finding on FDG-PET/CT scan.
